# {6,6′-Diethoxy-2,2′-[4,5-dimethyl-*o*-phenylenebis(nitrilomethylidyne)]di­phenolato}nickel(II) dihydrate

**DOI:** 10.1107/S1600536809012641

**Published:** 2009-04-08

**Authors:** Hadi Kargar, Reza Kia, Arezoo Jamshidvand, Hoong-Kun Fun

**Affiliations:** a Department of Chemistry, School of Science, Payame Noor University (PNU), Ardakan, Yazd, Iran; b X-ray Crystallography Unit, School of Physics, Universiti Sains Malaysia, 11800 USM, Penang, Malaysia

## Abstract

In the title complex, [Ni(C_26_H_26_N_2_O_4_)]·2H_2_O, the Ni^II^ ion, lying on a twofold crystallographic rotation axis, has a square-planar geometry, being coordinated by the N_2_O_2_ unit of the tetra­dentate Schiff base ligand. The asymmetric unit of the title compound comprises one-half of the complex mol­ecule and one of the water mol­ecules of crystallization. The water H atoms form bifurcated O—H⋯(O,O) hydrogen bonds with the O atoms of the phenolato and eth­oxy groups with *R*_1_^2^(5) and *R*_1_^2^(6) ring motifs. The dihedral angle between the central benzene ring and the two outer benzene rings are 4.07 (11) and 3.99 (12)°. The dihedral angle between the two O–Ni–N coordination planes is only 0.77 (11)°. In the crystal structure, the mol­ecules are linked together into extended chains along the *c* axis by inter­molecular O—H⋯O and C—H⋯O inter­actions. An inter­esting feature of the crystal structure is a short inter­molecular C ⋯ C [3.355 (3) Å] contact, which is shorter than the sum of the van der Waals radii. The crystal structure may be further stabilized by inter­molecular π–π inter­actions [centroid–centroid distances in the range 3.5758 (13)–3.6337 (13) Å].

## Related literature

For bond-length data, see Allen *et al.* (1987 ▶). For related structures see, for example: Clark *et al.* (1968 ▶, 1969 ▶, 1970 ▶). For the applications and bioactivity of Schiff base complexes see, for example: Elmali *et al.* (2000 ▶); Blower (1998 ▶); Granovski *et al.* (1993 ▶); Li & Chang, (1991 ▶); Shahrokhian *et al.* (2000 ▶). For hydrogen-bond motifs, see: Bernstein *et al.* (1995 ▶).
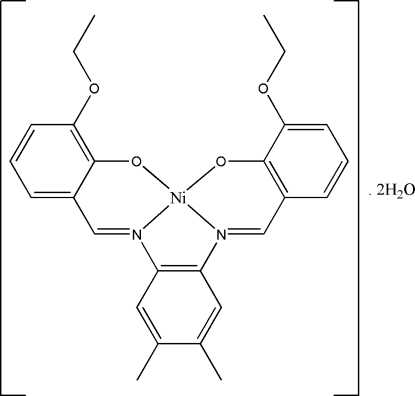


## Experimental

### 

#### Crystal data

[Ni(C_26_H_26_N_2_O_4_)]·2H_2_O
*M* *_r_* = 525.23
Orthorhombic, 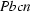

*a* = 12.8706 (4) Å
*b* = 16.1130 (4) Å
*c* = 11.8546 (3) Å
*V* = 2458.45 (12) Å^3^
*Z* = 4
Mo *K*α radiation
μ = 0.83 mm^−1^
*T* = 294 K
0.30 × 0.16 × 0.08 mm

#### Data collection

Bruker APEXII CCD area-detector diffractometer
Absorption correction: multi-scan (**SADABS**; Bruker, 2005 ▶) *T*_min_ = 0.790, *T*_max_ = 0.935
16330 measured reflections
3517 independent reflections
2007 reflections with *I* > 2σ*I*)
*R*_int_ = 0.065

#### Refinement

*R*[*F* ^2^ > 2σ(*F* ^2^)] = 0.046
*wR*(*F* ^2^) = 0.122
*S* = 1.01
3517 reflections
161 parameters
H-atom parameters constrained
Δρ_max_ = 0.29 e Å^−3^
Δρ_min_ = −0.51 e Å^−3^

### 

Data collection: *APEX2* (Bruker, 2005 ▶); cell refinement: *SAINT* (Bruker, 2005 ▶); data reduction: *SAINT*; program(s) used to solve structure: *SHELXTL* (Sheldrick, 2008 ▶); program(s) used to refine structure: *SHELXTL*; molecular graphics: *SHELXTL*; software used to prepare material for publication: *SHELXTL* and *PLATON* (Spek, 2009 ▶).

## Supplementary Material

Crystal structure: contains datablocks global, I. DOI: 10.1107/S1600536809012641/cs2114sup1.cif

Structure factors: contains datablocks I. DOI: 10.1107/S1600536809012641/cs2114Isup2.hkl

Additional supplementary materials:  crystallographic information; 3D view; checkCIF report

## Figures and Tables

**Table 1:** Selected bond lengths (Å)

Ni1—O1	1.8447 (15)
Ni1—N1	1.8573 (17)

**Table 2:** Hydrogen-bond geometry (Å, °)

*D*—H⋯*A*	*D*—H	H⋯*A*	*D*⋯*A*	*D*—H⋯*A*
O1*W*—H1*W*1⋯O1	0.82	2.50	3.087 (2)	129
O1*W*—H1*W*1⋯O2	0.82	2.39	3.145 (3)	152
O1*W*—H2*W*1⋯O1^i^	0.82	2.47	3.083 (3)	133
O1*W*—H2*W*1⋯O2^i^	0.82	2.41	3.121 (3)	146
C7—H7*A*⋯O1*W*^ii^	0.93	2.57	3.383 (3)	146

Symmetry codes: (i) 
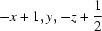
; (ii) 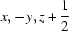.

**Fig. 1.**
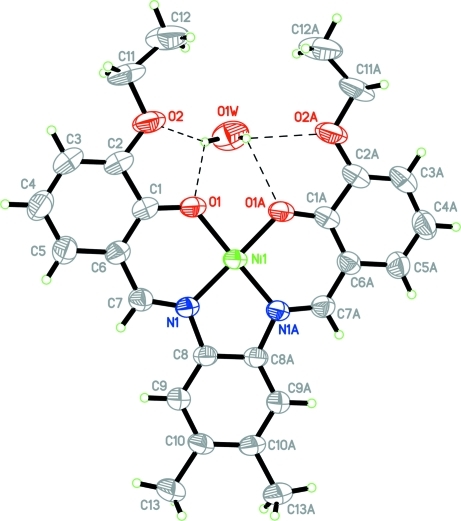
The title molecular compound, showing 50° probability displacement ellipsoids and the atomic numbering, hydrogen bonds are shown as dashed lines. Symmetry code for suffix A: -x + 1, y, -z + 1/2.

**Fig. 2.**
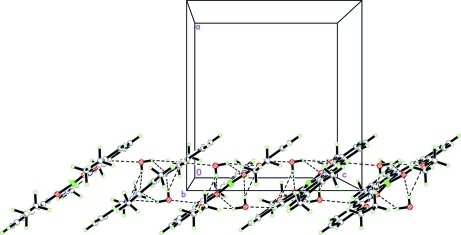
A crystal packing excerpt of the title compound viewed down the b-axis, showing 1-D extended chains along the c-axis. Intermolecular interactions are drawn as dashed lines.

## supplementary crystallographic information

### Comment

Schiff base complexes are some of the most important stereochemical models in transition metal coordination chemistry, with their ease of preparation and structural variations (Granovski *et al.*, 1993). Metal derivatives of Schiff bases have been studied extensively, and copper(II) and Ni(II) complexes play a major role in both synthetic and structural research (Elmali *et al.*, 2000; Blower, 1998; Granovski *et al.*, 1993; Li & Chang, 1991; Shahrokhian *et al.*, 2000). Tetradentate Schiff base metal complexes may form *trans* or *cis* planar or tetrahedral structures (Elmali *et al.*, 2000).

The Ni^II^ ion of the title compound (Fig. 1), shows a square planar geometry which is coordinated by two imine N atoms and two phenol O atoms of the tetradentate Schiff base ligand and lies across a crystallographic twofold rotation axis. The bond lengths (Allen *et al.*,, 1987) and angles are within normal ranges and are comparable with the related structures (Clark *et al.*, 1968, 1969, 1970). The water H atoms form bifurcated O—H(O,O) intermolecular hydrogen bonds with the oxygen atoms of the phenolato- and ethoxy groups with *R^2^_1_(5)* and *R^2^_1_(6)* ring motifs (Bernstein *et al.*, 1995), which may, in part, influence the molecular configuration (Fig. 1). The dihedral angle between the central benzene ring and the two outer benzene rings are 4.07 (11) and 3.99 (12)°. The dihedral angle between the two coordination planes O1—Ni1—N1 and O1A—Ni1—N1A is 0.77 (11)°. In the crystal structure the complex and two water molecules, association of which form the title compound, are linked together into 1-D extended chains by intermolecular O—H···O and C—H···O interactions along the *c* axis (Fig. 2). The interesting feature of the crystal structure is a short intermolecular C1···C7^iii^ [3.355 (3) Å; (iii) 1 - x, -y, 1 - z ] contact, shorter than the sum of the van der Waals radius of carbon atoms. The crystal structure is further stabilized by intermolecular π-π [*Cg*1···*Cg*3^iii^ = 3.5758 (13) Å; *Cg*2···*Cg*2^iii^ = 3.6085 (11) Å; *Cg*2···*Cg*3^iii^ = 3.6337 (13) Å, *Cg*1, *Cg*2 and *Cg*3 are the centroid of the Ni1/N1/C8/C8A/N1A, C1–C6, and Ni1/O1/C1/C6/C7/N1 rings, respectively].

### Experimental

A chloroform solution (40 ml) of [*N,N'*-Bis(3-ethoxy-salicylidene)- 4,5-dimethyl-phenylenediamine (1 mmol) was added to a ethanol solution (20 mL) of NiCl_2_.6H_2_O (1.05 mmol, 237 mg). The mixture was refluxed for 30 min and then filtered. After keeping the filtrate in air, deep-red block-shaped crystals were formed at the bottom of the vessel on slow evaporation of the solvent.

### Refinement

The water H-atoms were located from the difference Fourier map and constrained to refine with the carrier atom after O—H distance restraint of 0.82 (1) Å. The rest of the hydrogen atoms were positioned geometrically [C—H = 0.95–97 Å] and refined using a riding approximation model. A rotating-group model was used for the methyl groups.

### Crystal data

**Table:**

[Ni(C_26_H_26_N_2_O_4_)]·2H_2_O	*F*(000) = 1104
*M**_r_* = 525.23	*D*_x_ = 1.419 Mg m^−^^3^
Orthorhombic, *P**b**c**n*	Mo *K*α radiation, λ = 0.71073 Å
Hall symbol: -P 2n 2ab	Cell parameters from 2497 reflections
*a* = 12.8706 (4) Å	θ = 2.7–22.3°
*b* = 16.1130 (4) Å	µ = 0.83 mm^−^^1^
*c* = 11.8546 (3) Å	*T* = 294 K
*V* = 2458.45 (12) Å^3^	Block, red
*Z* = 4	0.30 × 0.16 × 0.08 mm

### Data collection

**Table:**

Bruker APEXII CCD area-detector diffractometer	3517 independent reflections
Radiation source: fine-focus sealed tube	2007 reflections with *I* > 2σ(*I*)
graphite	*R*_int_ = 0.065
φ and ω scans	θ_max_ = 29.8°, θ_min_ = 2.5°
Absorption correction: multi-scan (*SADABS*; Bruker, 2005)	*h* = −17→17
*T*_min_ = 0.790, *T*_max_ = 0.935	*k* = −22→22
16330 measured reflections	*l* = −16→11

### Refinement

**Table:**

Refinement on *F*^2^	Primary atom site location: structure-invariant direct methods
Least-squares matrix: full	Secondary atom site location: difference Fourier map
*R*[*F*^2^ > 2σ(*F*^2^)] = 0.046	Hydrogen site location: inferred from neighbouring sites
*wR*(*F*^2^) = 0.122	H-atom parameters constrained
*S* = 1.01	*w* = 1/[σ^2^(*F*_o_^2^) + (0.0543*P*)^2^ + 0.1108*P*] where *P* = (*F*_o_^2^ + 2*F*_c_^2^)/3
3517 reflections	(Δ/σ)_max_ < 0.001
161 parameters	Δρ_max_ = 0.29 e Å^−^^3^
0 restraints	Δρ_min_ = −0.51 e Å^−^^3^

### Fractional atomic coordinates and isotropic or equivalent isotropic displacement parameters (Å^2^)

**Table:**

	*x*	*y*	*z*	*U*_iso_*/*U*_eq_	
Ni1	0.5000	−0.00935 (2)	0.2500	0.04044 (15)	
O1	0.44372 (13)	0.07589 (9)	0.33400 (13)	0.0501 (4)	
O2	0.38481 (16)	0.21443 (10)	0.42373 (15)	0.0716 (6)	
N1	0.44336 (14)	−0.09376 (10)	0.33719 (15)	0.0398 (4)	
C1	0.38667 (17)	0.06930 (14)	0.4246 (2)	0.0441 (5)	
C2	0.3505 (2)	0.14350 (15)	0.4772 (2)	0.0524 (6)	
C3	0.2894 (2)	0.14174 (16)	0.5706 (2)	0.0605 (7)	
H3A	0.2659	0.1912	0.6020	0.073*	
C4	0.2614 (2)	0.06548 (19)	0.6203 (2)	0.0660 (8)	
H4A	0.2193	0.0646	0.6841	0.079*	
C5	0.2961 (2)	−0.00668 (16)	0.5746 (2)	0.0566 (7)	
H5A	0.2790	−0.0568	0.6088	0.068*	
C6	0.35820 (19)	−0.00679 (13)	0.4755 (2)	0.0451 (6)	
C7	0.39054 (17)	−0.08387 (14)	0.42982 (19)	0.0441 (5)	
H7A	0.3725	−0.1315	0.4695	0.053*	
C8	0.46786 (17)	−0.17483 (13)	0.29740 (18)	0.0412 (5)	
C9	0.43332 (19)	−0.24982 (14)	0.3410 (2)	0.0498 (6)	
H9A	0.3879	−0.2497	0.4020	0.060*	
C10	0.4649 (2)	−0.32468 (14)	0.2957 (2)	0.0519 (6)	
C11	0.3545 (2)	0.29234 (15)	0.4698 (3)	0.0759 (9)	
H11A	0.3783	0.2974	0.5471	0.091*	
H11B	0.2794	0.2979	0.4687	0.091*	
C12	0.4041 (3)	0.35795 (18)	0.3968 (3)	0.1078 (13)	
H12A	0.3824	0.4119	0.4219	0.162*	
H12B	0.3832	0.3500	0.3198	0.162*	
H12C	0.4783	0.3537	0.4022	0.162*	
C13	0.4247 (3)	−0.40558 (15)	0.3438 (2)	0.0791 (10)	
H13A	0.3822	−0.3943	0.4086	0.119*	
H13B	0.4823	−0.4399	0.3656	0.119*	
H13C	0.3841	−0.4338	0.2877	0.119*	
O1W	0.38489 (17)	0.20940 (12)	0.15851 (16)	0.0853 (6)	
H1W1	0.3725	0.1955	0.2239	0.128*	
H2W1	0.4475	0.2018	0.1648	0.128*	

### Atomic displacement parameters (Å^2^)

**Table:**

	*U*^11^	*U*^22^	*U*^33^	*U*^12^	*U*^13^	*U*^23^
Ni1	0.0477 (2)	0.0286 (2)	0.0450 (3)	0.000	0.00116 (19)	0.000
O1	0.0667 (11)	0.0307 (8)	0.0530 (10)	0.0029 (8)	0.0057 (9)	−0.0006 (7)
O2	0.1017 (15)	0.0353 (9)	0.0778 (13)	0.0146 (9)	0.0068 (11)	−0.0068 (9)
N1	0.0447 (11)	0.0313 (9)	0.0434 (11)	0.0007 (8)	0.0005 (9)	−0.0028 (8)
C1	0.0453 (13)	0.0388 (12)	0.0481 (14)	0.0059 (10)	−0.0064 (11)	−0.0071 (11)
C2	0.0561 (15)	0.0429 (14)	0.0582 (16)	0.0118 (12)	−0.0078 (12)	−0.0123 (12)
C3	0.0610 (17)	0.0553 (16)	0.0651 (18)	0.0144 (13)	−0.0023 (14)	−0.0211 (14)
C4	0.0646 (17)	0.0690 (19)	0.0643 (18)	0.0047 (14)	0.0141 (14)	−0.0177 (15)
C5	0.0582 (16)	0.0584 (16)	0.0533 (17)	−0.0020 (12)	0.0091 (13)	−0.0069 (12)
C6	0.0447 (13)	0.0418 (13)	0.0487 (15)	0.0012 (10)	−0.0008 (10)	−0.0074 (11)
C7	0.0470 (13)	0.0392 (12)	0.0461 (14)	−0.0042 (10)	0.0000 (11)	−0.0020 (10)
C8	0.0496 (13)	0.0312 (11)	0.0429 (13)	0.0000 (9)	−0.0010 (10)	−0.0010 (9)
C9	0.0603 (15)	0.0384 (12)	0.0507 (14)	0.0005 (11)	0.0115 (12)	0.0003 (11)
C10	0.0660 (16)	0.0321 (12)	0.0576 (15)	−0.0057 (10)	0.0056 (12)	0.0027 (10)
C11	0.079 (2)	0.0387 (15)	0.111 (2)	0.0179 (14)	−0.0129 (18)	−0.0251 (16)
C12	0.161 (4)	0.0409 (17)	0.122 (3)	0.016 (2)	−0.011 (3)	−0.0029 (18)
C13	0.111 (3)	0.0372 (15)	0.089 (2)	−0.0093 (15)	0.0313 (19)	0.0036 (14)
O1W	0.1029 (16)	0.0723 (14)	0.0809 (14)	0.0234 (12)	−0.0218 (12)	0.0009 (11)

### Geometric parameters (Å, °)

**Table:**

Ni1—O1^i^	1.8447 (15)	C7—H7A	0.9300
Ni1—O1	1.8447 (15)	C8—C9	1.387 (3)
Ni1—N1	1.8573 (17)	C8—C8^i^	1.396 (4)
Ni1—N1^i^	1.8573 (17)	C9—C10	1.381 (3)
O1—C1	1.306 (3)	C9—H9A	0.9300
O2—C2	1.380 (3)	C10—C10^i^	1.410 (5)
O2—C11	1.423 (3)	C10—C13	1.514 (3)
N1—C7	1.301 (3)	C11—C12	1.508 (4)
N1—C8	1.424 (2)	C11—H11A	0.9700
C1—C6	1.415 (3)	C11—H11B	0.9700
C1—C2	1.427 (3)	C12—H12A	0.9600
C2—C3	1.358 (3)	C12—H12B	0.9600
C3—C4	1.410 (4)	C12—H12C	0.9600
C3—H3A	0.9300	C13—H13A	0.9600
C4—C5	1.358 (3)	C13—H13B	0.9600
C4—H4A	0.9300	C13—H13C	0.9600
C5—C6	1.421 (3)	O1W—H1W1	0.8226
C5—H5A	0.9300	O1W—H2W1	0.8179
C6—C7	1.417 (3)		
			
O1^i^—Ni1—O1	83.75 (10)	N1—C7—H7A	117.2
O1^i^—Ni1—N1	178.81 (7)	C6—C7—H7A	117.2
O1—Ni1—N1	95.21 (7)	C9—C8—C8^i^	119.32 (13)
O1^i^—Ni1—N1^i^	95.21 (7)	C9—C8—N1	127.2 (2)
O1—Ni1—N1^i^	178.81 (7)	C8^i^—C8—N1	113.45 (11)
N1—Ni1—N1^i^	85.84 (11)	C10—C9—C8	121.4 (2)
C1—O1—Ni1	127.19 (14)	C10—C9—H9A	119.3
C2—O2—C11	117.8 (2)	C8—C9—H9A	119.3
C7—N1—C8	120.49 (18)	C9—C10—C10^i^	119.14 (14)
C7—N1—Ni1	125.80 (15)	C9—C10—C13	120.3 (2)
C8—N1—Ni1	113.61 (14)	C10^i^—C10—C13	120.54 (14)
O1—C1—C6	124.54 (19)	O2—C11—C12	106.4 (2)
O1—C1—C2	118.4 (2)	O2—C11—H11A	110.5
C6—C1—C2	117.1 (2)	C12—C11—H11A	110.5
C3—C2—O2	125.3 (2)	O2—C11—H11B	110.5
C3—C2—C1	121.8 (2)	C12—C11—H11B	110.5
O2—C2—C1	112.9 (2)	H11A—C11—H11B	108.6
C2—C3—C4	120.5 (2)	C11—C12—H12A	109.5
C2—C3—H3A	119.8	C11—C12—H12B	109.5
C4—C3—H3A	119.8	H12A—C12—H12B	109.5
C5—C4—C3	119.7 (3)	C11—C12—H12C	109.5
C5—C4—H4A	120.2	H12A—C12—H12C	109.5
C3—C4—H4A	120.2	H12B—C12—H12C	109.5
C4—C5—C6	121.1 (2)	C10—C13—H13A	109.5
C4—C5—H5A	119.5	C10—C13—H13B	109.5
C6—C5—H5A	119.5	H13A—C13—H13B	109.5
C1—C6—C7	121.4 (2)	C10—C13—H13C	109.5
C1—C6—C5	119.8 (2)	H13A—C13—H13C	109.5
C7—C6—C5	118.8 (2)	H13B—C13—H13C	109.5
N1—C7—C6	125.7 (2)	H1W1—O1W—H2W1	93.7
			
O1^i^—Ni1—O1—C1	−177.9 (2)	C2—C1—C6—C7	−179.8 (2)
O1—Ni1—N1—C7	−4.77 (19)	O1—C1—C6—C5	179.7 (2)
N1^i^—Ni1—N1—C7	175.8 (2)	C2—C1—C6—C5	0.0 (3)
O1—Ni1—N1—C8	178.82 (14)	C4—C5—C6—C1	1.5 (4)
N1^i^—Ni1—N1—C8	−0.62 (11)	C4—C5—C6—C7	−178.6 (2)
Ni1—O1—C1—C6	0.5 (3)	C8—N1—C7—C6	−177.5 (2)
Ni1—O1—C1—C2	−179.89 (15)	Ni1—N1—C7—C6	6.3 (3)
C11—O2—C2—C3	0.2 (4)	C1—C6—C7—N1	−3.4 (4)
C11—O2—C2—C1	−179.2 (2)	C5—C6—C7—N1	176.7 (2)
O1—C1—C2—C3	178.9 (2)	C7—N1—C8—C9	6.6 (4)
C6—C1—C2—C3	−1.4 (3)	Ni1—N1—C8—C9	−176.77 (19)
O1—C1—C2—O2	−1.6 (3)	C7—N1—C8—C8^i^	−174.9 (2)
C6—C1—C2—O2	178.1 (2)	Ni1—N1—C8—C8^i^	1.8 (3)
O2—C2—C3—C4	−178.1 (2)	C8^i^—C8—C9—C10	3.0 (4)
C1—C2—C3—C4	1.3 (4)	N1—C8—C9—C10	−178.6 (2)
C2—C3—C4—C5	0.3 (4)	C8—C9—C10—C10^i^	1.0 (5)
C3—C4—C5—C6	−1.7 (4)	C8—C9—C10—C13	−179.1 (2)
O1—C1—C6—C7	−0.2 (4)	C2—O2—C11—C12	178.9 (2)

Symmetry codes: (i) −*x*+1, *y*, −*z*+1/2.

### Hydrogen-bond geometry (Å, °)

**Table:**

*D*—H···*A*	*D*—H	H···*A*	*D*···*A*	*D*—H···*A*
O1W—H1W1···O1	0.82	2.50	3.087 (2)	129
O1W—H1W1···O2	0.82	2.39	3.145 (3)	152
O1W—H2W1···O1^i^	0.82	2.47	3.083 (3)	133
O1W—H2W1···O2^i^	0.82	2.41	3.121 (3)	146
C7—H7A···O1W^ii^	0.93	2.57	3.383 (3)	146

Symmetry codes: (i) −*x*+1, *y*, −*z*+1/2; (ii) *x*, −*y*, *z*+1/2.
